# Hybrid Solution Combining Kalman Filtering with Takagi–Sugeno Fuzzy Inference System for Online Car-Following Model Calibration

**DOI:** 10.3390/s20195539

**Published:** 2020-09-27

**Authors:** Mădălin-Dorin Pop, Octavian Proștean, Tudor-Mihai David, Gabriela Proștean

**Affiliations:** 1 Automation and Applied Informatics Department, Politehnica University of Timisoara, Bvd. V. Parvan, No. 2, 300223 Timisoara, Romania; madalin.pop@student.upt.ro; 2 Faculty of Automation and Computers, Politehnica University of Timisoara, Bvd. V. Parvan, No. 2, 300223 Timisoara, Romania; tudor.david@student.upt.ro; 3 Management Department, Politehnica University of Timisoara, Bvd. M. Viteazu, No. 1, 300223 Timisoara, Romania; gabriela.prostean@upt.ro

**Keywords:** fuzzy inference, calibration, car-following, Takagi–Sugeno, Kalman filter, microscopic traffic model, continuous-time model

## Abstract

Nowadays, the intelligent transportation concept has become one of the most important research fields. All of us depend on mobility, even when we talk about people, provide services, or move goods. Researchers have tried to create and test different transportation models that can optimize traffic flow through road networks and, implicitly, reduce travel times. To validate these new models, the necessity of having a calibration process defined has emerged. Calibration is mandatory in the modeling process because it ensures the achievement of a model closer to the real system. The purpose of this paper is to propose a new multidisciplinary approach combining microscopic traffic modeling theory with intelligent control systems concepts like fuzzy inference in the traffic model calibration. The chosen Takagi–Sugeno fuzzy inference system proves its adaptive capacity for real-time systems. This concept will be applied to the specific microscopic car-following model parameters in combination with a Kalman filter. The results will demonstrate how the microscopic traffic model parameters can adapt based on real data to prove the model validity.

## 1. Introduction

Traffic modeling is one of the research domains receiving a higher interest from scientists. Traffic congestion [1] affects everyone and introduces a dependence on this issue even if we consider the movement of people or the delivery of products or services. In this context, a concept has appeared regarding smart mobility that tries to use ITS (intelligent transportation systems)-specific algorithms to optimize road traffic flow.

In the traffic modeling process, there are several subsystems that contribute to the determination of a closer-to-reality road traffic model. For each new model proposal, a validation system ensures the comparison of those model simulation results and real traffic data. The calibration process will minimize the differences between simulation and real data. This calibration is responsible for the improvement in simulation parameters by ensuring a better fitting of them compared to real data.

The current paper aims to deliver a new calibration method for road traffic parameters at the microscopic level. This method consists of a Kalman filter integrated with a Takagi–Sugeno FIS (fuzzy inference system) for the car-following model in continuous-time. The Takagi–Sugeno FIS using the Kalman-filtered values as input for the car-following model consists of our calibration system proposal.

This paper starts with an overview of actual relevant research about road transportation improvements and fuzzy logic applications.

The third section of this paper presents a description of the car-following model, considered the most known microscopic road traffic model. After highlighting some general features of the calibration process, the paper introduces the application of a continuous-time Kalman filter for the online calibration of the car-following model.

Section 4 describes the FIS using the Kalman-filtered values as inputs for the relative velocity between FV (follower vehicle) and LV (leader vehicle) and simulates the inter-vehicle spacing estimation error relative to the measurement error. This FIS aims to provide the specific offset for inter-vehicle spacing until the system is calibrated.

The last three sections consist of a case study presentation, results analysis, discussions and conclusions related to our research.

## 2. Literature Overview

Recent trends in improvements in urban road traffic provide a direction of research to common platforms creation for the intelligent management of sensor networks, having as the main objective the assurance of appropriate real-time decisions in different environmental conditions [2]. To improve the sensor data collection and transmission, new technologies can be used such as 5G and VLC (visible-light communication) [3]. The energy consumption is one of the major current problems in road monitoring systems, and smart solutions for energy savings are welcomed [4].

Currently, ITS development involves the usage of Bluetooth sensors in the TMS (traffic monitoring systems). This approach applied in several cities can enhance their chance to become smart cities and, implicitly, to improve the citizens’ quality of life [5]. This sensing technology proved its utility in travel times estimation and traffic signal control. An interesting approach in this regard uses a modifiedmax-pressure algorithm based on travel times collection from Bluetooth sensors. The results from an experiment consisting of a real implementation of this approach in a signalized intersection from Jerusalem demonstrated its validity and brought many advantages. The benefits of this technology compared to video-based systems arose from the easier installation and maintenance processes and the availability of measurements of turning ratios [6]. On the same direction of Bluetooth technology usage, researchers propose in [7] a real-time road traffic monitoring system based on the purchased data from private companies. To improve the quality of these data, an augmentation framework that uses Bayesian inference is proposed and uses the ability of Bluetooth sensing technology to present the bimodal traffic flow pattern that ensures compliance with the speed limits. In this regard, an experiment on a principal arterial corridor in Orlando, USA achieved good results.

During the past few years, fuzzy logic has been finding a fast-growing number of applications in different fields [8]. The road traffic forecasting represents an interesting direction in ITS research. The usage of atmospheric and air pollution data for this reason in approaches based on recurrent neural networks [9] shows the high level of intelligent systems implications in solving road traffic problems. Another possible solution of traffic flow performance forecasting is to use fuzzy neural networks [10]. This approach of forecasting methods expects a wide mixture of factors like information from drivers that have an important impact on traffic concentration and capacity of the urban road network. 

The installation of the sensors appointed for traffic data collection is crucial for traffic lights management and traffic jam detection, and can bring improved travel times for the vehicles crossing the road network [11]. To achieve this goal of short travel times, a solution is to set the green and red intervals by using a fuzzy controller designed to dynamically update the maximum values associated with the green intervals [12]. Another approach is to use an adaptive traffic light system based on the fuzzy Q-learning control method using the intelligent agents concept [13]. 

A major problem related to the traffic flow is the determination and the control of the dynamic distance between two vehicles. A good approach in this regard is to use the fuzzy interpolation for distance-gap computation, considering that planning for collision has a pre-set constant time [14]. The use of automate driving can minimize this risk of collision, through a method that can determine an adaptive velocity for the vehicles participating in the traffic process. 

The driver behavior consists of one of the most challenging things in transportation modeling and represents the main source of uncertainty. Video surveillance systems prove their efficiency in road traffic patterns identification and behaviors classification. In this regard, the usage of a combination between the PAM (Pachinko allocation model) with the SVM (support vector machine) achieved better performances than the LDA (latent Dirichlet allocation) approach [15]. An innovative proposal shows that traffic patterns can be retrieved from social media applications that enable user geo-location data sharing. From this perspective, individual activities durations can be retrieved and correlated with the geo-location data to reconstruct the user mobility trajectories [16].

Besides the microscopic traffic models, there are two more types of models like macroscopic and mesoscopic. Macroscopic traffic models focus on the complete road flow, involving vehicle distributions and traffic density, and mesoscopic models consider the vehicles as vehicle groups in the modeling process. Regarding the calibration of the microscopic parameters, some researchers consider that this can be avoided by adopting the macroscopic modeling approach. In this case, the existence of an MFD (macroscopic fundamental diagram) can easily estimate the space-mean flows and trip completion rates. The experiment used fixed detectors and floating vehicles as sensors for data collection [17]. Nevertheless, a disadvantage of this macroscopic evaluation is a lack of inter-vehicle behavior evaluation that can provide individual driving patterns.

## 3. Microscopic Traffic Models Calibration

Microscopic traffic models [18] give more attention to the details of traffic flow and are vital for traffic analysis, especially in the presence of ITS. Initial model calibration is necessary to identify the parameter values. It requires the activities of all participants in traffic in order to have feedback of the traffic with parameters like vehicle position, accelerations/decelerations, and vehicle speed. Moreover, the interaction between the vehicles involved in the movement process needs special attention [19].

### 3.1. Car-Following Model—General Description

Car-following is a microscopic traffic model that analyzes how two vehicles interact during movement. One of these vehicles is the LV and the other is the FV. The second one shall adapt its movement parameters based on the vehicle ahead behavior [20]. Further, starting from MIMO (multiple-input multiple-output) systems theory, the paper presents the state-space representation of this road traffic model in continuous-time.

The inputs of the state-space system for the linear continuous model of the car-following are the vehicles’ accelerations/decelerations $u_{1}$ and $u_{2}$, and the standard safety distance between the vehicles S. The state of this system is represented by the vehicles’ velocities noted as $x_{1}$ and $x_{3}$, and the running distances of the vehicles, $x_{2}$ and $x_{4}$. Based on these, the system output y consisting of the dynamic safety distance between FV and LV will be computed and will be further used to adapt the FV parameters to ensure compliance with the established standard safety distance.

The linear continuous car-following model previously presented can be characterized using the following system equations, as it was stated in [21,22,23]:

(1) $$\{\begin{array}{l} \left[\begin{array}{c} {\dot{x}}_{1}\\ {\dot{x}}_{2}\\ {\dot{x}}_{3}\\ {\dot{x}}_{4} \end{array}\right]\;=\;\left[\begin{array}{cccc} 0 & 0 & 0 & 0\\ 1 & 0 & 0 & 0\\ 0 & 0 & 0 & 0\\ 0 & 0 & 1 & 0 \end{array}\right]\cdot \left[\begin{array}{c} x_{1}\\ x_{2}\\ x_{3}\\ x_{4} \end{array}\right]+\left[\begin{array}{cc} 1 & 0\\ 0 & 0\\ 0 & 1\\ 0 & 0 \end{array}\right]\cdot \left[\begin{array}{c} u_{1}\\ u_{2} \end{array}\right]\\ y\;=\;\left[\begin{array}{cccc} 0 & -1 & 0 & 1 \end{array}\right]\cdot [\begin{array}{c} x_{1}\\ x_{2}\\ x_{3}\\ x_{4} \end{array}]\;+\;S\; \end{array}$$

The standard safety distance S computation considers the vehicle average length L:

(2) $$S\;=\;L\cdot \left(1\;+\;\frac{x_{3}}{16.10}\right)$$

To obtain the classic structure of MIMO state-space representation, some notations according to (3) shall be defined:

(3) $$\;\begin{array}{l} {\bar{x}}_{1}{\;=\;x}_{3}\;-{\;x}_{1},\\ {\bar{x}}_{2}{\;=\;x}_{4}\;-{\;x}_{2}\;=\;s\\ \bar{y}\;=\;{\bar{x}}_{2} \end{array},$$

where s is the dynamic distance between LV and FV.

Rewriting (1) by using the notations from (3), the MIMO state-space representation of the linear continuous car-following model looks like:

(4) $$\{\begin{array}{l} \dot{\bar{x}}\left(t\right)\;=\;A\cdot \bar{x}\left(t\right)\;+\;B\cdot u\left(t\right)\\ \bar{y}\left(t\right)\;=\;C\cdot \bar{x}\left(t\right)\;+\;S \end{array}$$

The vectors and matrices extended forms are: $\bar{x}\;=\;\left[\begin{array}{c} {\bar{x}}_{1}\\ {\bar{x}}_{2} \end{array}\right]$, $u\;=\;\left[\begin{array}{c} u_{1}\\ u_{2} \end{array}\right]$, $A\;=\;\left[\begin{array}{cc} 0 & 0\\ 1 & 0 \end{array}\right]$, $B\;=\;\left[\begin{array}{cc} -1 & 1\\ 0 & 0 \end{array}\right]$, and $C\;=\;\left[\begin{array}{cc} 0 & 0\\ 0 & 1 \end{array}\right]$. Moreover, the system eigenvalues equal zero, the concatenated matrix $\left[\begin{array}{cc} A & B \end{array}\right]$ is controllable, and the matrix $\left[\begin{array}{cc} A & C \end{array}\right]$ is observable.

### 3.2. Calibration Process

The calibration process finds many issues in the case of microscopic traffic modeling. One of the common problems is the measurement of traffic characteristics like velocity, travel times, and the distance between vehicles because of the influence introduced by each vehicle behavior or travel conditions [19]. The calibration data shall consider this uncertainty in driver decisions and need to adapt to it.

**Figure 1:**
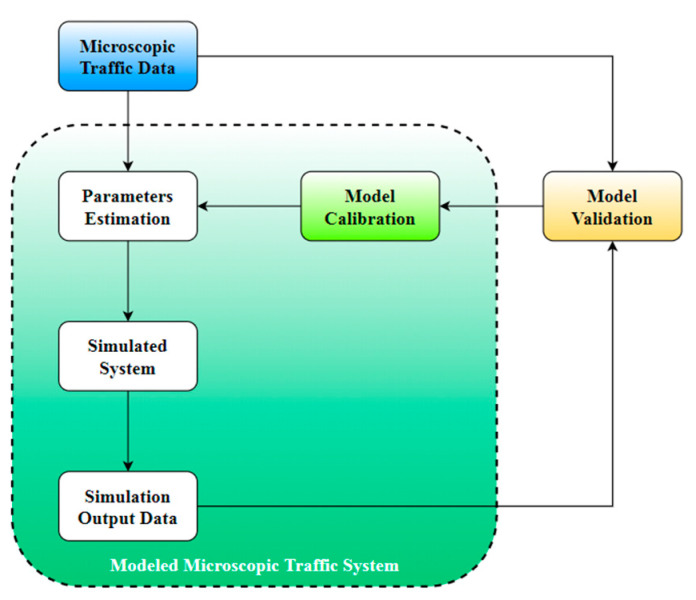
Microscopic traffic system.

Figure 1 illustrates a description of road traffic systems at the microscopic level. The following three main components ensure the existence of a model closer to reality: microscopic traffic data, modeled microscopic traffic system, and model validation. 

The first component is responsible for collecting traffic data from the real world. The second component simulates the real system behavior and uses the estimated values for road traffic parameters as inputs to calculate the simulation output data. A comparison between the simulated data and the real microscopic traffic data consists of a validation step. The output of this component is a decision based on the similarity between the real and simulated model and consists of the calibration component input. The calibration step shall establish the offset values to be applied to the model inputs to reduce the difference compared to the real data. Calibration is performed until these offset values become equal to zero.

### 3.3. Kalman Filter for Online Calibration

Online calibration is specific for systems that are using real-time data in order to adapt the model, by changing the simulated parameters to be close to the real system. This category of calibration includes the Kalman filter method [24,25,26].

Kalman filters are popular in the calibration of state-space models characterizing a system in discrete-time, but also applies to continuous-time systems [27], as in our case. For the microscopic road traffic, this method proved its efficiency even if the traffic conditions were nonstationary or stationary. This approach permits a real-time continuous update of studied parameters by updating them through the specific offsets. Further, the application of this method will be presented for car-following model calibration [26].

The state equations for the calibration system of the car-following model according to the Kalman filtering concept are shown in (5), where $\gamma_{i}\left(t\right),\;i\;=\;\left\{1,\;3,\;s\right\}$ are the parameters that need calibration.

(5) $$\{\begin{array}{l} {\dot{x}}_{1}\left(t\right){\;=\;x}_{1}\left(t\right){\;+\;\gamma }_{1}\left(t\right)\\ {\dot{x}}_{3}\left(t\right){\;=\;x}_{3}\left(t\right){\;+\;\gamma }_{3}\left(t\right)\\ \dot{s}\left(t\right)\;=\;s\left(t\right)\;+\;\left(x_{1}\left(t\right)\;{-\;x}_{3}\left(t\right)\right)\cdot {T\;+\;\gamma }_{s}\left(t\right) \end{array}$$

Considering that $\zeta_{i}\left(t\right),\;i\;=\;\left\{1,\;3,\;s\right\}$ are the measurement errors for the real traffic parameters, the output equations of the calibration system are computed by using relation (6).

(6) $$\{\begin{array}{l} x_{1}^{\mathrm{obs}}\left(t\right){\;=\;x}_{1}\left(t\right){\;+\;\zeta }_{1}\left(t\right)\\ x_{3}^{\mathrm{obs}}\left(t\right){\;=\;x}_{3}\left(t\right){\;+\;\zeta }_{3}\left(t\right)\\ s^{\mathrm{obs}}\left(t\right)\;=\;s\left(t\right){\;+\;\zeta }_{s}\left(t\right) \end{array}$$

Based on previous assumptions, the Kalman filter state-space representation can be obtained as follows:

(7) $$\{\begin{array}{l} \dot{x}\left(t\right){\;=\;A}_{k}\cdot x\left(t\right){\;+\;B}_{k}\cdot u\left(t\right){\;+\;D}_{k}\cdot \gamma \left(t\right)\\ y\left(t\right){\;=\;C}_{k}\cdot x\left(t\right)\;+\;\zeta \left(t\right) \end{array}$$

where the matrices specific for the continuous Kalman filter have the following values: $A_{k\;}=\;\left[\begin{array}{ccc} 1 & 0 & 0\\ 0 & 1 & 0\\ T & -T & 1 \end{array}\right]$, $B_{k}\;=\;\left[0\right]$, $C_{k}\;=\;\left[\begin{array}{ccc} 1 & 0 & 0\\ 0 & 1 & 0\\ 0 & 0 & 1 \end{array}\right]$, and $D_{k}\;=\;\left[\begin{array}{ccc} 1 & 0 & 0\\ 0 & 1 & 0\\ 0 & 0 & 1 \end{array}\right]$.

The continuous-time Kalman filter estimates the values according to (8):

(8) $$\hat{x}\left(t\right)\;=\;{\hat{x}}_{\mathrm{pr}}\left(t\right){\;+\;K}_{k}\cdot (y\left(t\right)\;-{\;C}_{k}\cdot {\hat{x}}_{\mathrm{pr}}\left(t\right))$$

where ${\hat{x}}_{\mathrm{pr}}\left(t\right)$ is a prediction obtained based on the previous knowledge using the following relation:

(9) $${\dot{\hat{x}}}_{\mathrm{pr}}\left(t\right){\;=\;A}_{k_{t-1}}\cdot {\hat{x}}_{\mathrm{pr}}\left(t\right){\;+\;B}_{k_{t-1}}\cdot {\hat{x}}_{\mathrm{pr}}\left(t\right)$$

where $A_{k_{t-1}}$ and $A_{k_{t-1}}$ represent the values of $A_{k}$ and $B_{k}$ at time t−1, respectively.

The gain matrix $K_{k}$ incorporates the compromise to adjust the estimated parameters by using real measured data but, at the same time, to avoid the propagation of measurement errors:

(10) $$K_{k}{\;=\;P}_{{\mathrm{pr}}_{k}}\;\cdot {\;C}_{k}^{T}\cdot {\left[C_{k}\cdot {\;P}_{{\mathrm{pr}}_{k}}\cdot {\;C}_{k}^{T}{\;+\;R}_{k}\right]}^{-1}$$

The matrix $P_{{\mathrm{pr}}_{k}}$ is the covariance matrix that contains the estimation error and will be updated at each step by applying the following relation:

(11) $$P_{{\mathrm{pr}}_{k+1}}{\;=\;A}_{k}\cdot {\;P}_{F_{k}}\cdot {\;A}_{k}^{T}{\;+\;D}_{k}\cdot {\;Q}_{k}\cdot {\;D}_{k}^{T}$$

where $P_{{\mathrm{pr}}_{k+1}}$ represents the value of $P_{{\mathrm{pr}}_{k}}$ at time t + 1, and $P_{F_{k}}$ is defined as:

(12) $$P_{F_{k}}\;=\;\left[{I\;-K}_{k}\cdot C_{k}\;\right]\;\cdot {\;P}_{{\mathrm{pr}}_{k}}$$

The Kalman filter covariance matrix of errors $R_{k}$ has the definition from (13).

(13) $$R_{k}\;=\;\left[\begin{array}{ccc} \sigma_{x_{1}\left(t\right){,\;x}_{1}{\left(t\right)}_{\;}} & 0 & \sigma_{x_{1}\left(t\right),\;s{\left(t\right)}_{\;}}\\ 0 & \sigma_{x_{3}\left(t\right){,\;x}_{3}{\left(t\right)}_{\;}} & \sigma_{x_{3}\left(t\right),\;s{\left(t\right)}_{\;}}\\ \sigma_{s\left(t\right){,\;x}_{1}\left(t\right){\;}_{\;}} & \sigma_{s\left(t\right){,\;x}_{3}\left(t\right){\;}_{\;}} & \sigma_{s\left(t\right),\;s{\left(t\right)}_{\;}} \end{array}\right]$$

with the propagation of variances and covariances expressed as:

(14) $$\sigma_{x_{n}s_{\;}}\;=\;\sum_{i,\;j}\frac{\partial x_{n}}{\partial x_{i}}\cdot \frac{\partial s}{\partial x_{j}}\cdot \sigma_{x_{\mathrm{ij}}{}_{\;}}$$

for the functions of interests defined as: $x_{n}\;=\;\left(x_{1}{,\;x}_{2},\;\cdots {,\;x}_{i},\;\cdots \right)$ and $s\;=\;\left(x_{1}{,\;x}_{2},\;\cdots {,\;x}_{j},\;\cdots \right)$, with the explanation that $\sigma_{x_{n}s_{\;}}$ is the covariance of $x_{n}$ and s, and the term $\sigma_{x_{\mathrm{ij}}}$ represents the covariance of $x_{i}$ and $x_{j}$.

## 4. Fuzzy Calibration of Microscopic Traffic Models

The FIS usually solves problems related to the nonlinearity of systems or in the cases of systems that have time delays, but is also suitable for continuous-time systems [28].

Microscopic traffic modeling is a complex problem because of the uncertainty in drivers’ decisions for lane changes or for the acceleration/deceleration behavior. The driver behavior influences all corresponding dynamic traffic parameters, introducing, in some cases, a swap in role between LV and FV. These reasons make the microscopic traffic modeling problem suitable for implementation with FIS, especially for the calibration process where all mentioned uncertainties need filtering in order to establish the best offset values. The application of these values ensures the dynamical adaptation of the modeled system to the received real traffic conditions.

In the following, we will show the particularities of the FIS, especially on the use of Takagi–Sugeno. A new calibration method will be issued based on this theoretical background in combination with the Kalman filtering concept previously presented. Compared to the simple use of Kalman filters, this hybrid approach tries to cover the learning of patterns in an offset setting.

### 4.1. Takagi–Sugeno FIS

Probably the most known model to implement an FIS is to use the Takagi–Sugeno approach [29,30,31,32,33]. Similar to other fuzzy methods, this model description uses the fuzzy specific IF-THEN rules. These input–output associations of the nonlinear modeled system characterize the dynamics of each fuzzy rule by creating a linear system model.

A general Takagi–Sugeno FIS for continuous-time models can be described by the following fuzzy rules [29,30]:

(15) $${\mathrm{IF}\;z}_{1}\left(t\right){\;\mathrm{is}\;F}_{i1}\;\mathrm{AND}\;\cdots {\;\mathrm{AND}\;z}_{p}\left(t\right){\;\mathrm{is}\;F}_{\mathrm{ip}}\;\mathrm{THEN}\;\{\begin{array}{l} \dot{x}\left(t\right)\;=\;\left(A_{i}\;+\;\Delta A_{i}\right)\cdot x\left(t\right)\;+\;\left(B_{i}\;+\;\Delta B_{i}\right)\cdot u\left(t\right)\\ y\left(t\right)\;=\;\left(C_{i}\;+\;\Delta C_{i}\right)\cdot x\left(t\right) \end{array}$$

where the notations have the following meaning:

- $z_{j}\left(t\right),\;j\;=\;\left\{1,\;2,\;\cdots ,\;p\right\}$ are the premise variables;
- $F_{\mathrm{ij}}\left(t\right),\;i\;=\;\left\{1,\;2,\;\cdots ,\;r\right\},\;\mathrm{and}\;j\;=\;\left\{1,\;2,\;\cdots ,\;p\right\}$ represent the fuzzy sets;
- r is the number of defined fuzzy rules;
- $x\left(t\right)\in R^{n}\;$ is the state vector;
- $u\left(t\right)\in R^{m}$ is the input vector;
- $A_{i}\in R^{n\times n}$ is the state matrix;
- $B_{i}\in R^{n\times m}$ is the input matrix;
- $C_{i}\in R^{q\times n}$ is the output matrix where q is the number of output parameters;
- $\Delta A_{i},\;\Delta B_{i},\;\mathrm{and}\;\Delta C_{i}$ are the matrices that incorporate the uncertainties.

The previous assumptions lead to the inferred model expression:

(16) $$\{\begin{array}{l} \dot{x}\left(t\right)\;=\;\overset{r}{\underset{i=1}{\sum }}h_{i}\left(z\left(t\right)\right)\cdot \left(\left(A_{i}\;+\;\Delta A_{i}\right)\cdot x\left(t\right)\;+\;\left(B_{i}\;+\;\Delta B_{i}\right)\cdot u\left(t\right)\right)\\ y\left(t\right)\;=\;\overset{r}{\underset{i=1}{\sum }}h_{i}\left(z\left(t\right)\right)\cdot \left(C_{i}\;+\;\Delta C_{i}\right)\cdot x\left(t\right) \end{array}$$

where $h_{i}\left(z\left(t\right)\right)$ is the normalized grade of membership for each rule and is compliant with (17).

(17) $$\{\begin{array}{l} \overset{r}{\underset{i=1}{\sum }}h_{i}\left(z\left(t\right)\right)\;=\;1\\ {0\;<\;h}_{i}\left(z\left(t\right)\right)\;<\;1 \end{array}$$

These equations represent the fundamentals for the implementation of an optimized calibration method for the special case of car-following models.

### 4.2. Car-Following Online Calibration based on Hybrid Takagi–Sugeno FIS Combined with Kalman Filtering

**Figure 2:**
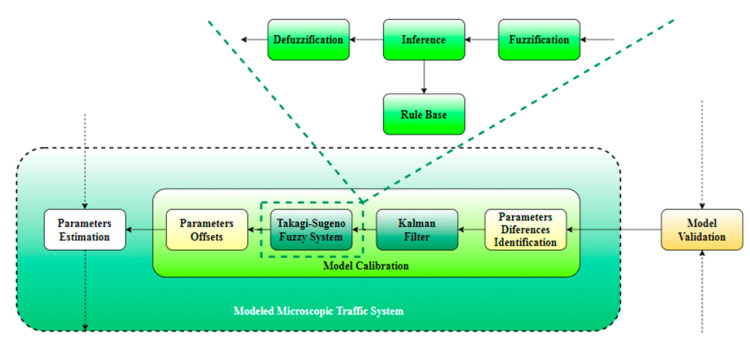
Proposed approach for microscopic traffic model calibration.

Figure 2 shows our proposal for the calibration system internal structure. Moreover, its relations with the other external subsystems are also represented. As part of the modeled microscopic traffic system, the calibration model has the feature of providing the necessary data to adapt the internal model parameters based on the evaluation received as input from the system responsible with the model validation. Together with the validation result, two sets of parameters consisting of simulation values and microscopic traffic real data will be sent to the calibration subsystem.

The first internal subsystem of a calibration subsystem is intended for identifying the differences between real and simulation data. These will be forwarded to the Kalman filter. The filtered values will be forwarded to the final decision step regarding the offset values consisting of a Takagi–Sugeno FIS. This last subsystem embeds the fuzzy specific components. The fuzzification ensures that the identified differences between the real and simulation parameters are converted to a fuzzy variable. Further, after the fuzzy rules have been defined, the output fuzzy variables will consider them in establishing the connections with the input fuzzy variables through an inference step. Defuzzification will convert the fuzzy variables to the corresponding types of analyzed parameters. These values consisting of offsets that shall be applied to the simulation parameters will also be saved by a subsystem that will build a knowledge base for the parameters offset.

After the model parameters have been updated with the offsets provided by the calibration subsystem, the process will be resumed until the offsets tend to zero. As the values of the simulation are closer to the real ones, the chances of the model to be validated increase. When the offsets are equal to zero, the model is considered as validated.

The inter-vehicle spacing offset for the next run of the simulation can be considered the same as the new estimated simulation error. From this point of view, the calibration module will be responsible for estimating the values that can reduce the difference between simulation values and the values retrieved from real traffic measurements. The new estimation error shall be computed as it is presented below and will be further applied directly by the subsystem that estimates the model parameters based on retrieved real traffic data, depending on corresponding conditioned parameters:

(18) $$\{\begin{array}{l} \Delta s\left(t\right)\;=\;\Delta s_{0}\left(t\right){\;+\;\zeta }_{s}\left(t\right)\;-{\;\gamma }_{s}\left(t\right),\;\gamma_{s}\left(t\right)\;\neq {\;\zeta }_{s}\left(t\right)\\ \Delta s\left(t\right)\;=\;\Delta s_{0}\left(t\right){\;+\;\gamma }_{s}\left(t\right)\;=\;\Delta s_{0}\left(t\right){\;+\;\zeta }_{s}\left(t\right),\;\gamma_{s}\left(t\right)\;=\;\zeta_{s}\left(t\right) \end{array}$$

where $\Delta s_{0}\left(t\right)$ is considered an initial offset that can be defined based on FV and LV behavior for velocity evolution, as can be seen in (19). This correction step shall be applied to ensure that it maintains the standard safety distance s between LV and FV:

(19) $$\Delta s_{0}\left(t\right)\;=\;\{\begin{array}{l} {0,\;x}_{1}\left(t\right)\;\geq {\;x}_{3}\left(t\right)\;\\ {S,\;x}_{1}\left(t\right){\;<\;x}_{3}\left(t\right) \end{array}$$

The model calibration will be done for the linguistic variables defined in Table 1. The inputs and outputs are defined in a manner that simplifies the fuzzy rules writing process. More than that, the output is defined for each possible situation that can describe the car-following model behavior from the LV and velocity evolution perspectives. Additional information that is taken into account is related to the measurement and simulation errors of dynamic safety distance between LV and FV (the inter-vehicle spacing). The output consisting of the necessary offsets that shall be applied to the simulation values for LV and FV vehicles will be defined by equations that use the Kalman filter-specific data. These offsets Δs will be computed according to (18).

Considering the introduced linguistic variables and the possible offset values defined by (18), the fuzzy rules are according to (20).

(20) $$\;\begin{array}{l} \mathrm{IF}\;\mathrm{REL}\_\mathrm{SPEED}\;=\;\mathrm{LOW}\;\;\mathrm{AND}\;\mathrm{REL}\_\mathrm{ERR}\;=\;\mathrm{LOW}\;\;\mathrm{THEN}\;\mathrm{OFFSET}\;=\;\mathrm{INCREASE}\;\\ \mathrm{IF}\;\mathrm{REL}\_\mathrm{SPEED}\;=\;\mathrm{LOW}\;\;\mathrm{AND}\;\mathrm{REL}\_\mathrm{ERR}\;=\;\mathrm{EQUAL}\;\mathrm{THEN}\;\mathrm{OFFSET}\;=\;\mathrm{MAINTAIN}\\ \mathrm{IF}\;\mathrm{REL}\_\mathrm{SPEED}\;=\;\mathrm{LOW}\;\;\mathrm{AND}\;\mathrm{REL}\_\mathrm{ERR}\;=\;\mathrm{HIGH}\;\;\mathrm{THEN}\;\mathrm{OFFSET}\;=\;\mathrm{REDUCE}\\ \mathrm{IF}\;\mathrm{REL}\_\mathrm{SPEED}\;=\;\mathrm{EQUAL}\;\mathrm{AND}\;\mathrm{REL}\_\mathrm{ERR}\;=\;\mathrm{LOW}\;\;\mathrm{THEN}\;\mathrm{OFFSET}\;=\;\mathrm{INCREASE}\\ \mathrm{IF}\;\mathrm{REL}\_\mathrm{SPEED}\;=\;\mathrm{EQUAL}\;\mathrm{AND}\;\mathrm{REL}\_\mathrm{ERR}\;=\;\mathrm{EQUAL}\;\mathrm{THEN}\;\mathrm{OFFSET}\;=\;\mathrm{MAINTAIN}\\ \mathrm{IF}\;\mathrm{REL}\_\mathrm{SPEED}\;=\;\mathrm{EQUAL}\;\mathrm{AND}\;\mathrm{REL}\_\mathrm{ERR}\;=\;\mathrm{HIGH}\;\;\mathrm{THEN}\;\mathrm{OFFSET}\;=\;\mathrm{REDUCE}\\ \mathrm{IF}\;\mathrm{REL}\_\mathrm{SPEED}\;=\;\mathrm{HIGH}\;\;\mathrm{AND}\;\mathrm{REL}\_\mathrm{ERR}\;=\;\mathrm{LOW}\;\;\mathrm{THEN}\;\mathrm{OFFSET}\;=\;\mathrm{INCREASE}\\ \mathrm{IF}\;\mathrm{REL}\_\mathrm{SPEED}\;=\;\mathrm{HIGH}\;\;\mathrm{AND}\;\mathrm{REL}\_\mathrm{ERR}\;=\;\mathrm{EQUAL}\;\mathrm{THEN}\;\mathrm{OFFSET}\;=\;\mathrm{MAINTAIN}\\ \mathrm{IF}\;\mathrm{REL}\_\mathrm{SPEED}\;=\;\mathrm{HIGH}\;\;\mathrm{AND}\;\mathrm{REL}\_\mathrm{ERR}\;=\;\mathrm{HIGH}\;\;\mathrm{THEN}\;\mathrm{OFFSET}\;=\;\mathrm{REDUCE} \end{array}$$

The fuzzy matrix $F_{k}$ that considers the fuzzy rules and (19) is shown in (21).

(21) $$F_{k}\;=\;\left[\begin{array}{ccc} {S\;+\;\zeta }_{s}\left(t\right){\;-\;\gamma }_{s}\left(t\right) & {S\;+\;\gamma }_{s}\left(t\right) & {S\;+\;\zeta }_{s}\left(t\right){\;-\;\gamma }_{s}\left(t\right)\\ \zeta_{s}\left(t\right){\;-\;\gamma }_{s}\left(t\right) & \gamma_{s}\left(t\right) & \zeta_{s}\left(t\right){\;-\;\gamma }_{s}\left(t\right)\\ \zeta_{s}\left(t\right){\;-\;\gamma }_{s}\left(t\right) & \gamma_{s}\left(t\right) & \zeta_{s}\left(t\right){\;-\;\gamma }_{s}\left(t\right) \end{array}\right]$$

## 5. Simulation and Results

This section describes the model implementation using Simulink from MATLAB R2020a. A case study for a real crossroad from Timișoara (Romania) will validate the proposed approach for car-following model calibration. 

### 5.1. Simulation Model

**Figure 3:**
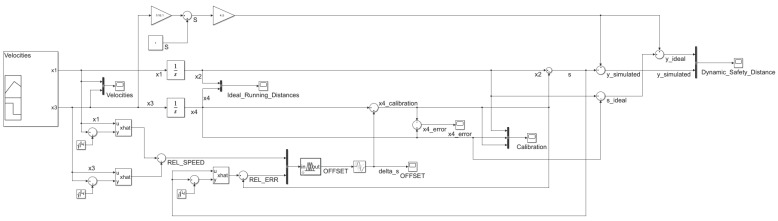
Simulation model—implementation using Simulink (MATLAB R2020a).

Figure 3 shows the Simulink (MATLAB R2020a) implementation model. The implementation is a simplified car-following model that studies the impact of velocities in the FV strategy to adapt its running distance based on the FV moving behavior. In this case, the acceleration was not taken into account.

The computation of the standard safety distance S is done continuously considering Equation (2). This value is needed to ensure collision avoidance in the case of the increase in FV velocity. For this computation, an average vehicle length L = 4.50 m was used. The output of the modeled system also considers S in the dynamic safety distance profile.

The inputs of the model and also the internal states can be affected by noise in the case of model implementation. To simulate this behavior, a Band-Limited White Noise Simulink block was added to the simulation for the velocities and inter-vehicle spacing to see their impact on the running distances for the LV and FV, and the dynamic safety distance y. The applied noise consists of normally distributed random numbers with the following characteristics that are the default values for the mentioned Simulink block:

- noise power: 0.10;
- sample time: 0.10;
- seed: 23341.

An adaptive system based on Kalman filtering and Takagi–Sugeno FIS will ensure the removal of the introduced noises by the simulated model. The Fuzzy Controller Simulink block uses the Kalman-filtered values as input for the relative speed between the FV and LV and also the simulated inter-vehicle spacing estimation error relative to the measurement error. The implementation details of the Takagi–Sugeno FIS are available in the second part of the current section.

Scope Simulink blocks were added in some points of interest for the following reasons:

- to monitor the input values;
- to obtain the running distances profile for the real behavior of the LV and FV;
- to capture the influences in the simulated running distances introduced by the simulated system;
- to correlate the calculated offset values for inter-vehicle spacing with the calibration process evolution;
- to create an overview of the calibration process impact on the system output.

#### 5.1.1. Input Data

**Figure 4:**
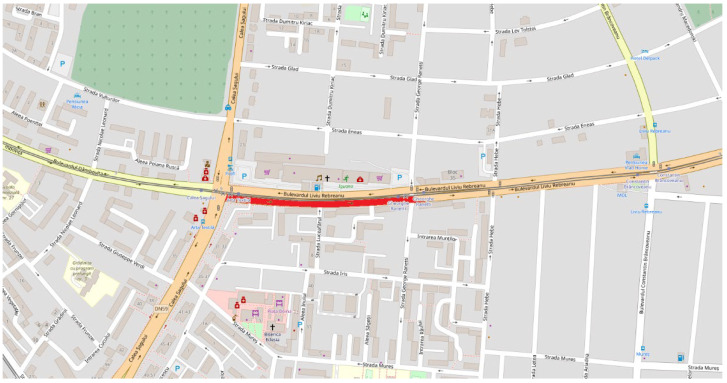
Real mapping of the studied piece of road (Source: *Open Street Map* view).

To prove the proposed model validity, the simulation uses, as input data, the data provided by Timișoara City Hall-General Directorate of Roads, Bridges, Parking and Utility Networks-Traffic Monitoring Office, Timișoara, Romania. Figure 4 shows, marked in red, the chosen piece of road between two crossroads: Liviu Rebreanu-Calea Șagului and Liviu Rebreanu-Gheorghe Ranetti. These crossroads have a high daily traffic flow and consists of a good input for our study. The data were collected by using inductive loop sensors that were placed on the studied road network to monitor the vehicle numbers and the velocities.

#### 5.1.2. Takagi–Sugeno Model Implementation Details

**Figure 5:**
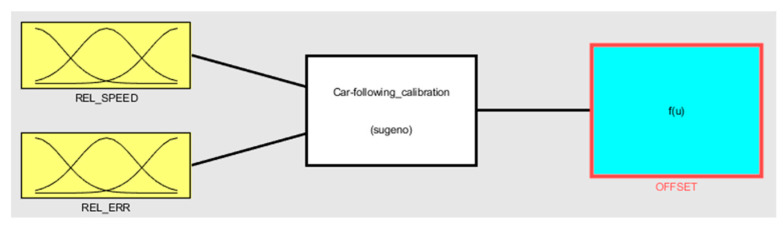
Takagi–Sugeno FIS calibration model implementation using Kalman-filtered values as input.

The implementation Takagi–Sugeno FIS using the Kalman-filtered values as inputs, according to the assumptions from the previous section, uses *Fuzzy Logic Toolbox* from MATLAB R2020a. Figure 5 shows the implemented calibration step as it looks in the chosen simulation tool.

**Figure 6:**
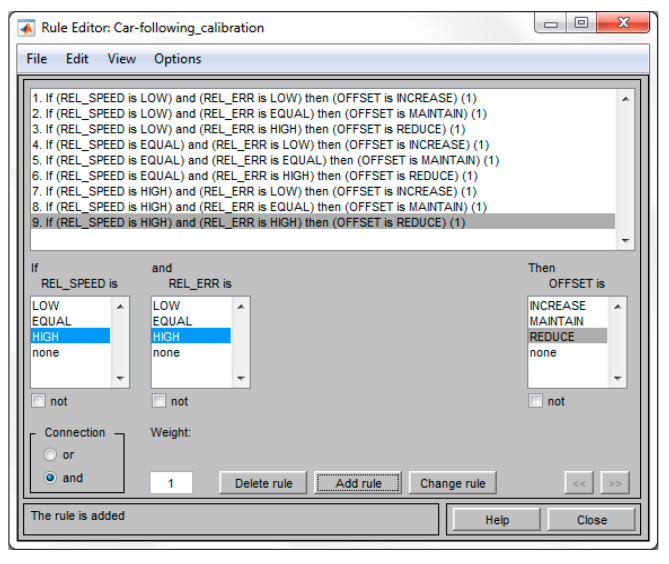
Fuzzy rules implementation using rule editor.

The established fuzzy rules presented in (20) were implemented for the car-following model calibration using the rule editor for Takagi–Sugeno FIS provided by the simulation tool, as it can be seen in Figure 6.

**Figure 7:**
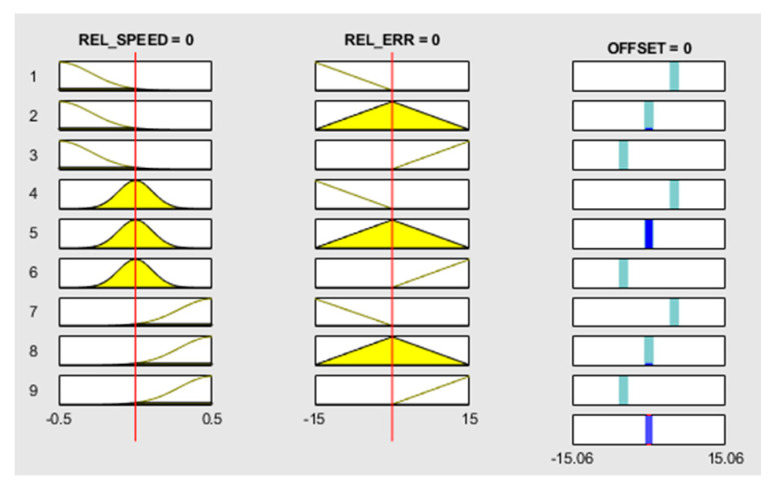
Rule viewer for Takagi–Sugeno FIS car-following calibration system.

A manner to analyze the simulation results specific to MATLAB R2020a is the usage of rule viewer. Each input of the calibration system and the output are shown in Figure 7. The output values of the car-following calibration system will be modified interactively based on chosen inputs. Membership functions were defined based on historical traffic data evolution.

**Figure 8:**
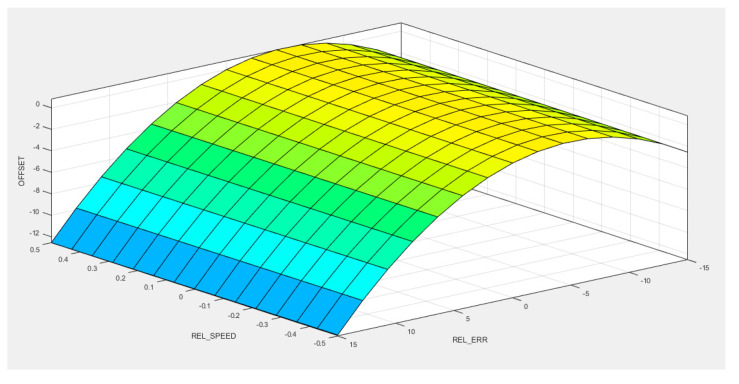
Surface viewer for Takagi–Sugeno FIS car-following calibration system.

Another option to analyze the results of fuzzy rules evaluation is through the surface viewer. Figure 8 illustrates the system performance for the relationship between the linguistic variables used as inputs or as output for the modeled microscopic traffic calibration system.

### 5.2. Simulation Results

**Figure 9:**
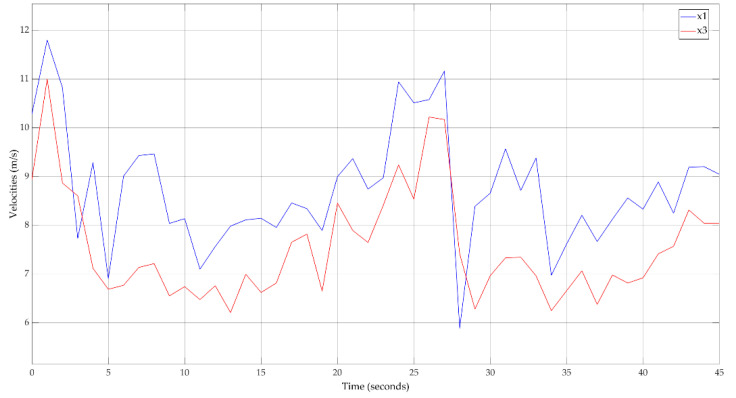
Input data—velocities for leader vehicle (LV) ($x_{1}$) and follower vehicle (FV) ($x_{3}$).

Figure 9 shows the input data for the proposed traffic calibration method. The evolution in time of the LV and FV velocities for the chosen piece of road between two intersections can be observed.

**Figure 10:**
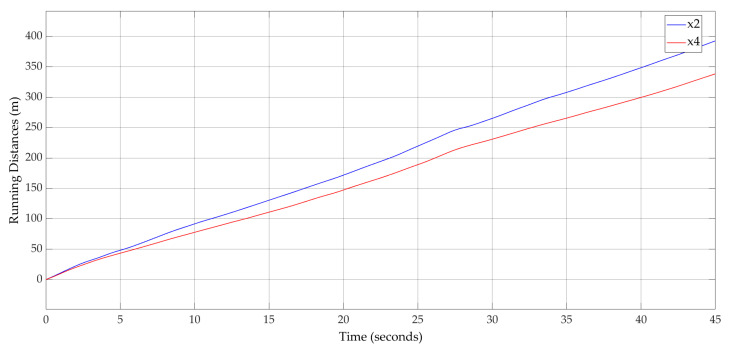
Running distances for LV ($x_{2}$) and FV ($x_{4}$)—ideal evolution.

Based on the input values, the ideal evolution was computed for the LV and FV running distances. Figure 10 shows this result after the application of the car-following approach for the real traffic data.

**Figure 11:**
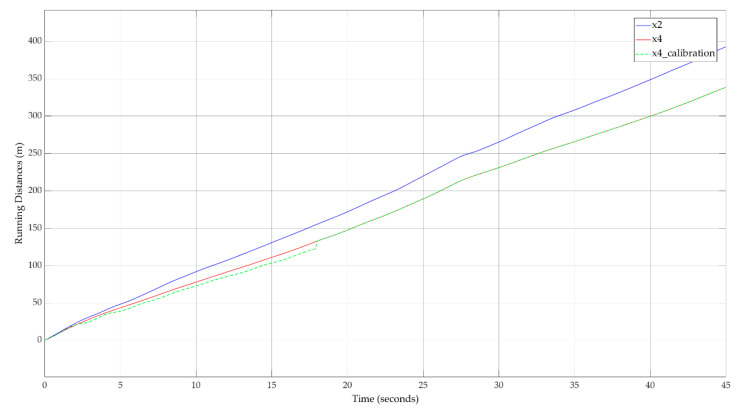
Running distances for LV ($x_{2}$) and FV ($x_{4}$)—calibration result for FV ($x_{4}$).

The result of the proposed hybrid approach for calibration of the car-following model in continuous-time is available in Figure 11. The modeled running distance of the FV that is affected by noise succeeds at reproducing the real behavior of the FV.

**Figure 12:**
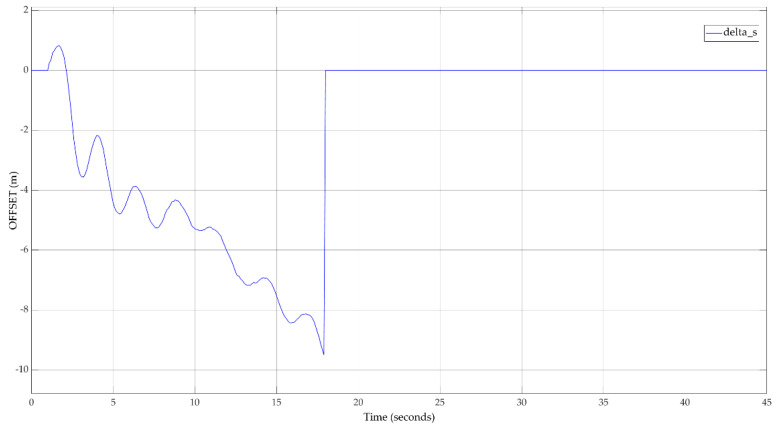
Inter-vehicle spacing offset applied to FV ($x_{4}$).

A better way to see the utility of the proposed approach is to analyze the calculated offset values. Figure 12 illustrates the evolution of the calculated offset values for the inter-vehicle spacing applied to the FV. Before the system joins the calibrated state, both positive and negative offset values are applied. After approximately t = 18 s, the system succeeded at learning different patterns of velocities and simulated inter-vehicle spacing error patterns and could reproduce the real behavior. After that time, the system was calibrated and could reproduce the real behavior.

**Figure 13:**
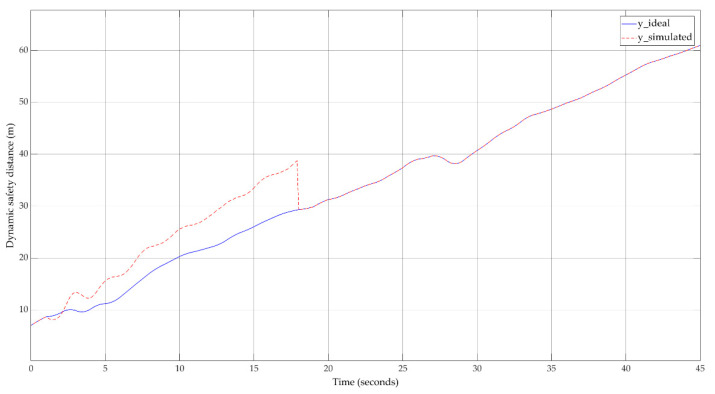
Dynamic safety distance—calibration overview.

The calibration result can also be observed by analyzing the dynamic safety distance that includes the standard safety distance. Figure 13 depicts the overview of the calibration result for the system output. The safety level is ensured by the direct application of safety length on the output, and safety is guaranteed by the car-following model even in cases of vehicles with different car lengths.

## 6. Discussions

**Figure 14:**
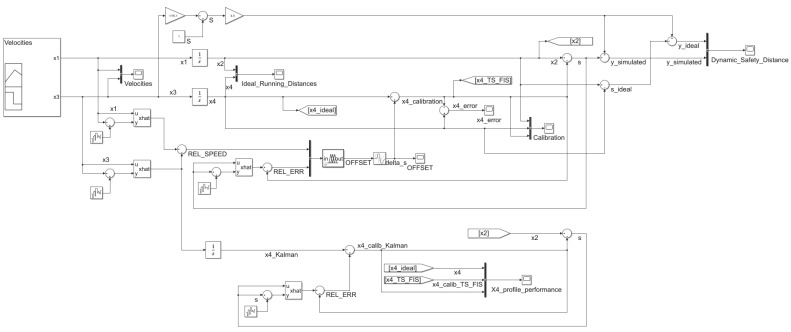
Simulation model to compare the Kalman filtering-only approach with the hybrid Kalman filtering and Takagi–Sugeno FIS approach-implementation using Simulink (MATLAB R2020a).

To show the motivation of using the hybrid proposal of Kalman filtering with Takagi–Sugeno FIS instead of a solution based on Kalman filtering only, a simulation is needed for this comparison. Figure 14 depicts the implementation using Simulink (MATLAB R2020a) for the mentioned reason. The simulation aims to analyze the behavior of FV for both cases of calibration methods. In addition to the simulation blocks from Figure 3, an extension introduces the computation of the FV running distance based on the Kalman filtering-only approach. In this case, the model uses only the filtered values to control the FV movement strategy. A big disadvantage, in this case, is the neglect of the FV relative velocity that can result in wrong offset values that will negatively influence the movement strategy. The calibration system shall ensure the capability for time-varying offset values application.

**Figure 15:**
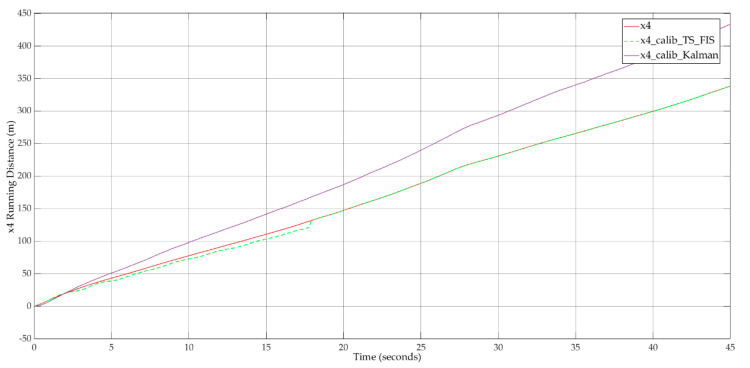
Running distance for FV ($x_{4}$—comparison of calibration result for the Kalman filtering-only approach and the hybrid Kalman filtering and Takagi–Sugeno FIS approach.

The simulation results for this comparison (Figure 15) illustrate that both approaches succeeded in filtering the noises and provided a similar-to-real-case trajectory evolution. The Kalman filtering-only approach cannot reproduce the real behavior through its neglect of inter-vehicle interaction from a relative velocity perspective. The hybrid approach takes advantage of this interaction between FV and LV and succeeds at identifying the time-varying appropriate offset value that reproduces the real behavior. This advantage can be visually observed from trajectory evolution where, after t = 18 s, the system is calibrated using the hybrid approach. In the same time, the Kalman filter-only approach introduces a uniform increase in computation error that leads to a scaled running distance compared to real traffic conditions. From a computational complexity perspective, both approaches fit to real-time processing. The Takagi–Sugeno FIS does not introduce computational delays that can lead to a major increase in the real-time data processing timings.

Another problem to address is the variety of vehicles with different lengths on the road network. The various vehicle lengths do not have a direct influence on the proposed method. The method intends to provide a calibration strategy based on the relative velocity and internal computed inter-vehicle spacings (s according to Equation (3)). The safety road assurance is done by a final step of adding a standard safety distance that depends on the LV length. The extension of this model for a chain of moving vehicles is already included even if we talk about pairs of vehicles where LV and FV are continuously changing. The data are retrieved from inductive loops sensors placed on the road network. The first vehicle that passes over the inductive loop is the LV and the second one is the FV. If we talk about a chain of vehicles that are moving on the road network, the FV from the first pair becomes the LV for the third vehicle and this behavior continues until the end of the chain of moving vehicles.

## 7. Conclusions

The purpose of this research was to propose a new approach of traffic models calibration at the microscopic modeling level. Our proposal consists of a combined solution that uses Kalman filtering and Takagi–Sugeno FIS for the online calibration of the car-following model.

A review of relevant works on ITS and fuzzy systems was needed to show the current trends in these research fields. Moreover, this created the necessary path to the introduction of our proposal.

A general presentation of the microscopic traffic modeling and calibration process, with special attention to the particular case of the car-following model, precedes our proposal. From an FIS perspective, our approach started with an introduction of the Takagi–Sugeno FIS followed with a mapping of the issues related to car-following models.

The proposed method was validated by the simulation results obtained from the Simulink (MATLAB R2020a) implementation of the continuous-time car-following model together with the system responsible for the calibration process. The input data of this study consisted of real road traffic data from Timișoara, Romania. Takagi–Sugeno FIS proved again its utility in adaptive systems through the optimization of the parameter offset-establishing process.

A comparison between a Kalman filtering-only approach and hybrid Kalman filtering with the Takagi–Sugeno FIS was conducted in Simulink (MATLAB R2020a). The comparison results show that the hybrid approach could provide a closer model to the real model. The big advantage of the hybrid approach was that it can provide the time-varying offset based on real-time road traffic parameters. Moreover, this proposal implies the specific interaction between FV and LV in the offset computation according to the microscopic traffic modeling theory.

Further works can extend this approach at the mesoscopic traffic modeling level where the conditions for velocities evaluation can be assigned to vehicle groups instead of individual vehicles. A big challenge in that direction will be the safety distance assurance inside a group of vehicles because mesoscopic modeling does not offer enough granularity compared to microscopic traffic models.

## Figures and Tables

**Table 1:** Linguistic variables for Takagi–Sugeno fuzzy inference system (FIS) based on Kalman-filtered values.

Parameter Role	Variable Name	Variable	Definition
Input 1	LV velocity relative to FV velocity (REL_SPEED)	LOW	$x_{1}\left(t\right){\;<\;x}_{3}\left(t\right)$
		EQUAL	$x_{1}\left(t\right){=x}_{3}\left(t\right)$
		HIGH	$x_{1}\left(t\right){\;>\;x}_{3}\left(t\right)$
Input 2	Simulated inter-vehicle spacing estimation error relative to measurement error (REL_ERR)	LOW	$\gamma_{s}\left(t\right)\;<\;\zeta_{s}\left(t\right)$
		EQUAL	$\gamma_{s}\left(t\right)=\;\zeta_{s}\left(t\right)$
		HIGH	$\gamma_{s}\left(t\right)\;>\;\zeta_{s}\left(t\right)$
Output	Inter-vehicle spacing offset Δs (OFFSET)	REDUCE	Equation (18)
		MAINTAIN	
		INCREASE	
